# Documentation of the evidence-diagnosis link predicts nutrition diagnosis resolution in the Academy of Nutrition and Dietetics' diabetes mellitus registry study: A secondary analysis of Nutrition Care Process outcomes

**DOI:** 10.3389/fnut.2023.1011958

**Published:** 2023-03-09

**Authors:** Casey Colin, Andrea Arikawa, Sherri Lewis, Melissa Cooper, Erin Lamers-Johnson, Lauri Wright, Constantina Papoutsakis

**Affiliations:** ^1^Department of Nutrition & Dietetics, University of North Florida, Jacksonville, FL, United States; ^2^James A. Haley Veterans' Hospital, Tampa, FL, United States; ^3^Academy of Nutrition and Dietetics, Chicago, IL, United States

**Keywords:** Type 2 diabetes, Type 1 diabetes, Nutrition Care Process, dietitians, medical nutrition therapy (MNT), outcomes

## Abstract

**Objectives:**

To describe nutrition care documentation patterns and investigate predictors of nutrition diagnosis resolution.

**Methods:**

This is a secondary data analysis of a 2-year pragmatic, quasi-experimental study conducted in outpatient clinics where nutrition care was provided to adults with diabetes Type 1 or 2 from May 2017 to June 2019 (*n* = 564 patients). The main outcome measures were frequency of standardized Nutrition Care Process (NCP) terms, NCP links, nutrition diagnosis resolution and predictors of nutrition diagnosis resolution. Predictors of diagnosis resolution were identified using a multivariable logistic regression model.

**Results:**

The most prevalent resolved diagnoses were excessive carbohydrate intake (32%), undesirable food choices (21%) and excessive energy intake (13%). The top etiology was food and nutrition related knowledge deficit (57%) and interventions were drawn mainly from the *Nutrition Education* domain (64%). One hundred forty-six patient cases (26%) had at least one follow-up visit and 26% of those with a follow-up (*n* = 38) had a resolved diagnosis. The presence of the evidence-diagnosis NCP link in documentation predicted diagnosis resolution (OR = 2.80, 95% CI 1.30–6.02; *p* = 0.008).

**Conclusion:**

Most diagnoses were caused by patients' lack of knowledge and respective interventions focused on nutrition education. Odds of diagnosis resolution improved when the signs and symptoms of the diagnosis were documented during assessment (evidence-diagnosis NCP link). Training dietitians on NCP links may be important to resolve nutrition diagnoses. Presented findings are hypothesis generating.

## 1. Introduction

Registered dietitian nutritionists (RDNs) apply medical nutrition therapy (MNT) to treat or manage nutrition diagnoses. A clearly defined connection between MNT and health improvement is necessary to demonstrate the contribution of MNT in improving patients' health. The Nutrition Care Process (NCP) model is the nutrition and dietetics profession's roadmap for nutrition care, and management of related results (1, 2). The NCP model has four steps and these are nutrition assessment and reassessment, diagnosis, intervention, and monitoring and evaluation. The first two steps identify the nutrition problem, and the last two solve or manage the identified problem. RDNs use standardized terminology to document nutrition care. This terminology is called the Nutrition Care Process Terminology (NCPT) and is an internationally adopted language that exceeds 2,000 terms. There is still a need to show the beneficial impact of MNT for specific health conditions. This can be done by pooling data to show care patterns and related patient outcomes.

**Figure 1:**
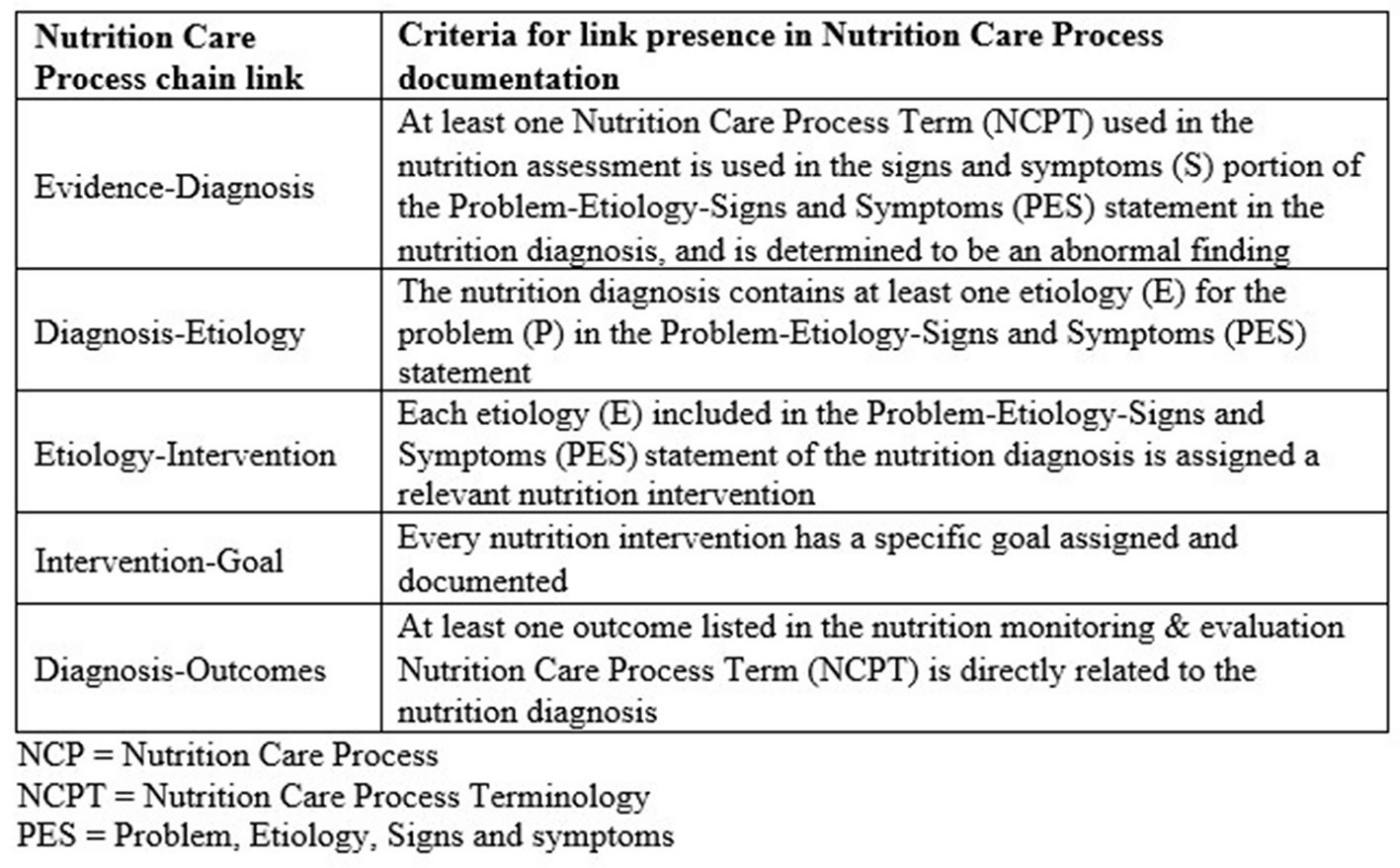
Criteria for determining presence of chain links in Nutrition Care Process (NCP) documentation (4). NCPT, Nutrition Care Process Terminology; PES, problem, the etiology, and the signs and symptoms.

An important aspect of NCP documentation includes creating logical links between the different components of the NCP model. There are five links between the six NCP components (evidence, diagnosis, etiology, goal, intervention, and outcome). All five links taken together are known as an NCP chain. NCP chains are a way to assess if the NCP was applied thoughtfully (2, 3). Murphy et al. (4) specified criteria for examining each of the chain links as part of the complete NCP chain (Figure 1). Specifically, to determine whether a complete NCP chain exists in documentation, one must examine the relevance of: the nutrition diagnosis given the assessment data; the etiology for the assessed nutrition diagnosis; the intervention in the context of the nutrition diagnosis etiology; the goals identified for the intervention; and the monitoring and evaluation data in the context of the nutrition diagnosis.

The application of the NCP is a required competence for RDNs. Yet there is a gap between intended and actual use of the NCP (5–7). Inconsistent documentation and poor application of standardized language make it difficult to demonstrate associations between nutrition interventions and outcomes. A research priority of the Academy of Nutrition and Dietetics is to evaluate the impact of nutrition care in various conditions such as diabetes mellitus and identify opportunities for improvement. The purpose of this study was to describe the documented nutrition care patterns and investigate predictors of nutrition diagnosis resolution in adults with diabetes mellitus Type 1 or 2 who received MNT in an outpatient setting.

## 2. Methods

The Diabetes Registry Study, is a pragmatic quasi-experimental study, that took place between May 2017 and June 2019. Specifically, routine nutrition care provided by RDNs to adult outpatients with diabetes mellitus Type 1 or 2 was documented for a randomly selected subset of patients. Documentation of nutrition care data was aggregated using the Academy of Nutrition and Dietetics Health Informatics Infrastructure (ANDHII) (3). ANDHII is a web-based platform where de-identified patient NCP data is collected for analyses (4).

The American Academy of Family Physicians Institutional Review Board reviewed the study protocol (#17-287) and determined the project was not research involving human subjects based on Office for Human Research Protections Guidance on Research Involving Coded Private Information or Specimens (8).

**Figure 2:**
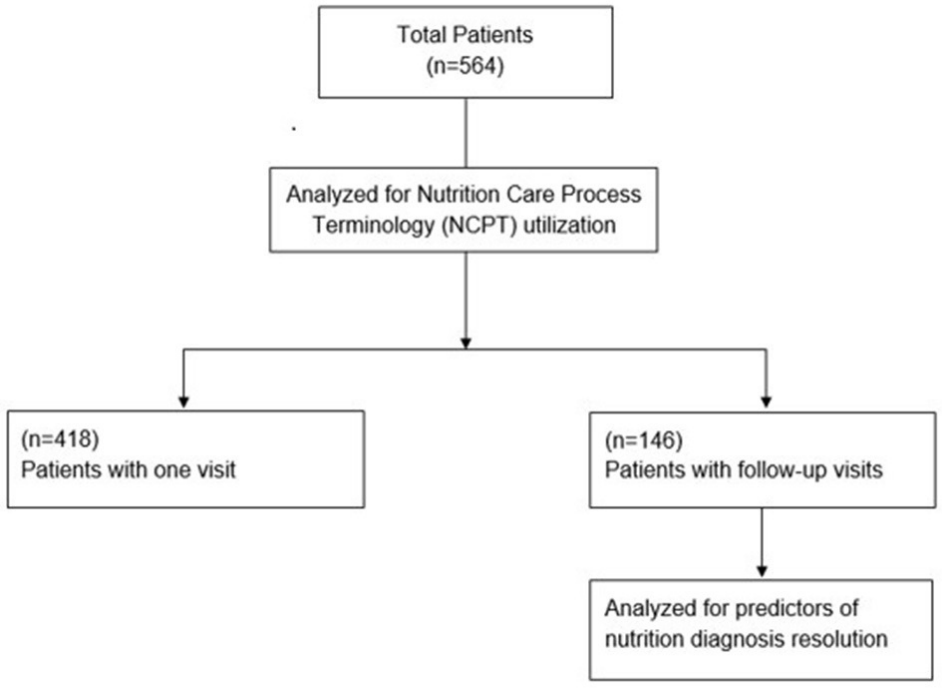
Data analysis flowchart for patients from the diabetes registry study.

Patient inclusion criteria were age >18 years, first outpatient visit to RDN for diabetes (patient may have received MNT for other conditions previously), and referral diagnosis for diabetes (Type 1, Type 2). Exclusion criteria were the following: diabetes was not listed on referral sheet as diagnosis, or patient had been treated by an RDN for diabetes previously. In 2017, an open call invitation was sent *via* email to members of the Academy of Nutrition and Dietetics Diabetes Practice Group and Nutrition Research Network. Sites were included when they had RDNs on staff who (1) responded to the call, (2) regularly provided outpatient nutrition care to adult patients with diabetes, and (3) completed a one-time in-person 5-h study orientation. Once the orientation was complete, data on patients who visited the site clinic were collected. The de-identified data from 564 patients aggregated from 22 different sites are analyzed in the present study as a secondary data analysis. Of these, 418 patients attended a single nutrition visit and 146 patients attended an initial nutrition visit and at least one follow up visit for MNT (Figure 2).

### 2.1. NCP terminology

The frequency of used NCP terms was determined. The nutrition diagnosis is a documentation statement made up of three parts: the problem (P), the etiology (E), and the signs and symptoms (S). The nutrition diagnosis is frequently referred to as the PES statement. In the NCP, indicators are variables tracked for improved results. For example, waist circumference is an indicator that may be tracked for change. Indicators were directly extracted from the s*igns and symptoms* portion of the PES statement. Indicators were assessed for goal progress only in patients who had one or more follow-up visits (*n* = 146). Positive goal progress was considered when the indicator's value either remained the same or improved. In some cases, indicators were not tracked between the initial and follow-up visits, therefore goal progress was unknown. Nutrition diagnosis resolution was defined as follows: a patient's nutrition problem was counted as resolved only when a status label of “resolved” was documented in the patient's note.

### 2.2. NCP chain links

Documentation from every patient was assessed for NCP chain links against the criteria defined by Murphy et al. (4) (Figure 1). A score of “yes” or “no” regarding link presence was awarded for each of the five NCP chain links. The NCP links are the “evidence-diagnosis” link; the “diagnosis-etiology” link; the “etiology-intervention” link; the “intervention-goal” link; and the “diagnosis-outcomes” link (Figure 1). Goals were identified either in the nutrition intervention section of the note, or in the monitoring and evaluation section of the note.

### 2.3. Statistical analysis

IBM SPSS Statistics version 26.0 (9) was used for statistical analyses, and statistical significance was set at *p* < 0.05. Descriptive statistics were expressed as frequency (percentage) for categorical variables and mean (standard deviation) for continuous and discrete variables. To evaluate predictors of diagnosis resolution a univariate analysis was carried out to examine relationships between putative predictive factors and the dependent variable (diagnosis resolution). Variables whose relationship was significant in the univariate analysis (*p* < 0.10) were entered into multivariable, backward stepwise logistic regression.

## 3. Results

The dataset of the Diabetes Registry Study contained NCP data on 790 total patient visits from 564 patients. Of the 564 patients, 418 had one visit without follow-up, leaving 146 patients (with a total of 372 visits including all follow-up visits). Nutrition diagnosis resolution was achieved in 26% of patients with follow up visits (38 of 146 patients).

### 3.1. NCP chain links

In patients with follow-up visits (*n* = 146), the evidence-diagnosis link had the lowest frequency (56%) compared to the other four links. The remaining link frequencies were: 97% for the diagnosis-etiology link and the etiology-intervention link, 95% for the intervention-goal link, and 88% for the diagnosis-outcome link. Of the 146 patients with follow-up visits, exactly half the patients (73) had complete NCP chains (all links present).

### 3.2. NCPT utilization

The five most frequently used terms in all patients (*n* = 564) for each NCP category are listed in the Supplementary Table 1. The most frequent nutrition assessment term was *glycosylated hemoglobin measurement* (17% of nutrition assessment terms). The most frequent problem was *excessive carbohydrate intake* (37% of nutrition diagnosis terms); the most frequent etiology was *food and nutrition related knowledge deficit* (57% of etiologies); and the most frequent sign and symptom was *glycosylated hemoglobin measurement* (18% of signs and symptoms). The most frequent nutrition intervention was *nutrition relationship to health/disease* (12% of nutrition intervention terms); and the most frequent nutrition monitoring and evaluation term was *glycosylated hemoglobin measurement* (17% of nutrition monitoring and evaluation terms).

### 3.3. Reassessment findings

Positive goal progress was achieved in 44% of goals (*n* = 282 goals from 146 patients). Also, goal progress was not achieved in 15% of goals. In some patients, indicators were not tracked from visit to visit, therefore goal progress was unknown (41% of goals). For example, in visit 1 fasting glucose was tracked for progress but then in visit 2 body weight was tracked and fasting glucose was not documented in visit 2. Thus, goal progress of fasting glucose could not be determined.

Nutrition diagnosis resolution could be assessed only in patients who had at least one follow-up visit (*n* = 146). Nutrition diagnosis resolution was not achieved in 74% (*n* = 108) of patients with follow up. Among patients that achieved nutrition diagnosis resolution (*n* = 38, 26%), the most frequently tracked indicator was *glycosylated hemoglobin measurement*, and the most common etiology category and nutrition intervention domain was *knowledge*, and *nutrition education*, respectively (Table 1).

**Table 1:** Distribution of Nutrition Care Process (NCP) standardized terminology used for patients with Type 1 and Type 2 diabetes mellitus that achieved nutrition diagnosis resolution (*n* = 38 patients).

**Resolution (*n*)**	**NCP term**	**Domain^a^**	**Frequency used *n* (%)**
**Nutrition diagnosis**			
Problem (*n* = 38 terms)	Excessive carbohydrate intake	Intake	12 (31.57)
	Undesirable food choices	Behavioral-environmental	8 (21.05)
	Excessive energy intake	Intake	5 (13.16)
	Food and nutrition related knowledge deficit	Behavioral-environmental	3 (7.89)
	Excessive fat intake^b^	Intake	2 (5.26)
Etiology (*n* = 38 terms)	Food and nutrition related knowledge deficit	Knowledge	27 (71.05)
	Disordered eating pattern	Psychological	2 (5.26)
	Not ready for diet/lifestyle change	Beliefs-attitudes	2 (5.26)
	Excessive carbohydrate intake^b^	Behavior	1 (2.63)
	Hyperglycemia^b^	Physiologic-metabolic	1 (2.63)
Signs and symptoms^c^ (*n* = 123 terms)	Glycosylated hemoglobin measurement	Biochemical data	36 (29.27)
	Glucose, fasting	Biochemical data	13 (10.57)
	Total carbohydrate intake	Intake	11 (8.94)
	Body weight	Anthropometric measurements	7 (5.69)
	Total energy intake	Intake	6 (4.88)
Nutrition intervention (*n* = 278 terms)	Recommended nutrition modifications	Nutrition education	47 (16.91)
	Nutrition relationship to health/disease	Nutrition education	33 (11.87)
	Other nutrition education	Nutrition education	31 (11.15)
	Physical activity guidance	Nutrition education	22 (7.91)
	Priority modifications, nutrition education	Nutrition education	19 (6.83)

a Etiology uses a category rather than a domain.

b When frequencies were equal among the next highest-used term, terms were listed in alphabetical order.

c igns and symptoms were the indicators used to determine goal progress, and the top 5 indicators with positive goal progress are listed here.

### 3.4. Predictors of diagnosis resolution

In univariate analysis, the diagnosis-etiology link (*p* = 0.749), the etiology-intervention link (*p* = 0.476), and the total number of visits (*p* = 0.974) were not related to diagnosis resolution and were not included in the multivariable regression model. The evidence-diagnosis link (*p* = 0.008) was significantly associated with diagnosis resolution and was included in the logistic regression model. The Intervention-goal link (*p* = 0.073) and the diagnosis-outcome link (*p* = 0.064) were not significantly related to diagnosis resolution but were included in the multivariable regression model. When all relevant predictors were entered into the multivariable regression model, the evidence-diagnosis link (*p* = 0.008) remained a significant predictor of diagnosis resolution. Documentation of the NCP evidence-diagnosis link increased the odds of diagnosis resolution significantly (OR = 2.80, 95% CI 1.30–6.02; *p* = 0.008; Table 2).

**Table 2:** Results of a backward, stepwise, multivariable analysis for predictors of nutrition diagnosis resolution in patients with Type 1 and Type 2 diabetes mellitus (*n* = 146).

**Variable**	***B***	**OR (95% CI)**	***p*-value**
Evidence-diagnosis link	1.03 (0.29)	2.80 (1.30, 6.02)	**0.008**
Constant	−0.57 (0.29)	0.21	< 0.001

Goodness of fit: [χ^2^ (1) = 7.23, p = 0.007]. Nagelkerke R^2^ = 0.07.

B, beta coefficient (standard error); OR, odds ratio; CI, confidence interval.

The *p*-value was bolded to emphasize the significance.

## 4. Discussion

To our knowledge, nutrition care documentation patterns according to the NCP model are described for the first time in outpatient adults with diabetes. The nutrition diagnosis resolution rate reached 26% in the Diabetes Registry Study. There was a preponderance of nutrition knowledge problems that were addressed with nutrition education interventions. At the patient level, documentation of the evidence-diagnosis NCP link improved the odds of nutrition diagnosis resolution (OR = 2.80, 95% CI 1.30–6.02; *p* = 0.008). These findings are hypothesis generating and support the need to research at a larger scale the contribution of NCP application in improved care.

### 4.1. NCPT utilization

The nutrition assessment data justify the presence of the nutrition diagnosis. Here, the most frequently used nutrition diagnosis was *excessive carbohydrate intake* (Table 1); yet assessment terms describing carbohydrate intake accounted for <3% (data not shown). Presence of the evidence-diagnosis link was a predictor of problem resolution. Lewis et al. (10) found that the etiology-intervention link was the most significant predictor of diagnosis resolution, and the evidence-diagnosis link was the second most significant predictor of problem resolution. In this study, the etiology-intervention link was not found to be a predictor of problem resolution (*p* = 0.476). This is a secondary data analysis and an a priori power analysis is not possible. However, the possibility of a type II error cannot be excluded in this instance. Murphy et al. (4) found that “evidence-initiated NCP chains” were significantly associated with complete NCP chains. Evidence-initiated chains are those chains that correctly include in the assessment documentation those data points that constitute the evidence (signs and symptoms) in the PES statement. We cannot be certain if the evidence-diagnosis link is a marker of better understanding of the NCP. Regardless of the mechanism, this finding is important for practitioners because it is linked to improved diagnosis resolution.

### 4.2. Nutrition intervention effectiveness

The most frequent etiology, *food and nutrition related knowledge deficit* (Table 1), should indicate that a lack of knowledge was observed. Yet <3% of assessment terms were knowledge-based (data not shown), which may indicate insufficient time (perceived or actual) for critical thinking to determine an accurate etiology for the problem(s) observed. The intervention may be appropriately directed toward resolving the documented etiology; but, if the lack of knowledge was not assessed, or the true etiology is not lack of knowledge, the intervention may not be appropriate. While the etiology-intervention link was present in 97% of follow-up cases, diagnosis resolution was observed in 26% of the cases. This misalignment supports the possibility of an inaccurately documented etiology. Another distinct possibility is that more nutrition care visits are needed to resolve nutrition diagnoses. A major characteristic of the Diabetes Registry Study was limited follow-up since only a quarter of cases were seen more than once.

Few interventions were from the counseling domain (data not shown), and most were from the education domain (Table 1). This finding is consistent with another study by Chui and colleagues (11), where authors noted a “heavy focus” on interventions from the education domain, and far less interventions from counseling. However, in a machine learning analysis of the same dataset that Chui and colleagues analyzed, counseling interventions were associated with higher diagnosis resolution rates when compared to interventions from other domains (12). Although counseling skills, including behavior change facilitation, are required by RDNs, these methods appear not to be used readily; or, if they are, they are not being documented (11). The NCPT includes terminology to describe counseling techniques and strategies. Yet, education terms are far more frequently documented compared to counseling, even when there is no evidence of knowledge deficit in the assessment. It is not clear if there is a need to improve RDNs' counseling competencies or RDNs are unfamiliar with the NCPT and do not utilize appropriate counseling terms. Further research is needed to understand why counseling terms are less frequently used.

### 4.3. Implications for practice

Nutrition care registry studies may serve as a valuable testing ground where gaps in existing terminology and terminology usage can be identified. Future registry studies should incorporate a way to report the reason for lack of follow-up nutrition visits (e.g., missed scheduled appointment). Understanding reasons for lack of follow up would help researchers to explore barriers that contribute to low outpatient attendance. Future studies should explore if increased counseling interventions have a positive impact on nutrition diagnosis resolution rate and/or number of return visits.

Advocating for an update in MNT financial coverage by the Centers for Medicare & Medicaid Services (CMS) has been on the forefront. The current reimbursement guidelines for diabetes in the US put limitations on the total number of MNT hours per year for Medicare beneficiaries, for example (13). In the spirit of holistic, individualized diabetes management, the number and frequency of visits should be patient driven. To solidify the platform on which to present a case for revision of MNT reimbursement legislation, more data should be available.

Collectively, the inconsistencies in NCP application reported here may indicate either inadequate understanding of NCP chain links, incomplete documentation for what is really happening in the MNT session, inadequate time for comprehensive MNT or charting, or inadequate NCP training overall. The possibility that continuous improvement in NCP terminology will help reduce such inconsistencies in documentation cannot be ruled out. It is important to also acknowledge that at present, entering data into ANDHII leads to double charting because ANDHII is not able to exchange data with other electronic health records. This fundamental information technology function is known as interoperability. The lack of ANDHII's interoperability comes with a time cost, and for busy clinicians, time is at a premium. Making RDNs aware of the NCP use and its importance is not enough for adoption (14). Lack of time (15), lack of motivation to change (16), and difficulties embracing change (17), are among the biggest reported barriers to using the NCP. However, use of the NCP increased physician support of nutrition care up to 90% (18). A major difference between RDNs and other allied health professions is that several allied health professions have been able to link objectively patient outcomes with the care they provide (19, 20).

In an era where digital learning prevails, providing state-of-the-art training on quality documentation is of paramount importance. Training and refresher courses should include understanding of NCP chain links, continuous outcomes tracking and overall quality application of the NCP.

### 4.4. Limitations

A strength of this study was that it exposed the large amount of broken NCP chains as a documentation issue and identified that intact chain links are associated with diagnosis resolution.

Several limitations exist within this study. RDNs who participated may be more enthusiastic about the NCP and NCPT compared to RDNs who did not participate. Thus, participation bias cannot be excluded as a possibility. ANDHII does not include a dedicated section on “goal,” so the interpretation of what the RDN considered a “goal” vs. what the researchers flagged as a “goal” may differ. This study examined documentation of MNT including documentation of counseling. Interpretations about MNT delivery including provision of limited counseling are not derived from observations of actual sessions. Frequency of follow up was tracked but not follow up time. The lack of follow up visits was high, goal progress was frequently not documented, and continuity in tracking indicators was problematic in patients with follow up. Positive goal progress included indicators which stayed the same, which may have artificially inflated the data if the indicator was the same solely due to lack of updated data (e.g., no new hemoglobin A1c available, so the previous result was documented). The NCP chain link evaluation has potential for the introduction of bias, as interpretation of the criteria often requires clinical judgement. Research results were limited by the available data of the Diabetes Registry Study. Nutrition diagnosis status labels must be unambiguous. Yet, available nutrition diagnosis status labels did not differentiate whether an active diagnosis was “improving,” or “not improving.” The Diabetes Registry Study is a pragmatic quasi experimental study that describes care under real life conditions. In future studies, the inclusion of a control group (patients who did not receive MNT from an RDN) is optimal. A power calculation was not performed prior to the Diabetes Registry Study. Given these limitations, results from this secondary data analysis may not be generalizable.

## 5. Conclusion

In this secondary data analysis of the Diabetes Registry Study, nutrition knowledge problems were the most frequently recorded problems, and these were managed with nutrition education. Also, there is a need for additional follow up given that only about a quarter of patients had documented follow up care and resolution of nutrition diagnoses was achieved 26% of times. At the patient level, the presence of the evidence-diagnosis NCP link improved the odds of nutrition diagnosis resolution. These predictive results are hypothesis generating. Additional studies are needed to replicate findings with larger samples.

## Data availability statement

The raw data supporting the conclusions of this article will be made available by the authors, without undue reservation.

## Author contributions

CC conducted the data analysis, with MC assisting in NCP audit scoring. CC wrote the first draft of the manuscript with input from LW, CP, AA, SL, and EL-J. All authors reviewed and commented on subsequent drafts of the manuscript.

## References

[1] Swan WI Vivanti A Hakel-Smith NA Hotson B Orrevall Y Trostler N. Nutrition care process and model update: toward realizing people-centered care and outcomes management. J Acad Nutr Diet. (2017) 117:2003–14. 10.1016/j.jand.2017.07.015 28988837

[2] Swan WI Pertel DG Hotson B Lloyd L Orrevall Y Trostler N. Nutrition Care Process (NCP) update part 2: developing and using the NCP terminology to demonstrate efficacy of nutrition care and related outcomes. J Acad Nutr Diet. (2019) 119:840–55. 10.1016/j.jand.2018.10.025 30660633

[3] Murphy WJ Steiber AL. A new breed of evidence and the tools to generate it: introducing ANDHII. J Acad Nutr Diet. (2015) 115:19–22. 10.1016/j.jand.2014.10.025 25534894

[4] Murphy WJ Yadrick MM Steiber AL Mohan V Papoutsakis C. Academy of Nutrition and Dietetics Health Informatics Infrastructure (ANDHII): a Pilot Study on the documentation of the Nutrition Care Process and the usability of ANDHII by registered dietitian nutritionists. J Acad Nutr Diet. (2018) 118:1966–1974. 10.1016/j.jand.2018.03.013 29804870

[5] Matthews KL Palmer MA Capra SM. The accuracy and consistency of Nutrition Care Process Terminology use in cases of refeeding syndrome. Nutr Diet. (2018) 75:331–6. 10.1111/1747-0080.12389 29114984

[6] O'Sullivan TA. Evaluation of an electronic record prototype incorporating the Nutrition Care Process and International Dietetics and Nutrition Terminology. Nutr Diet. (2013) 70:188–95. 10.1111/1747-0080.12012

[7] Enrione EB Reed D Myers EF. Limited agreement on etiologies and signs/symptoms among registered dietitian nutritionists in clinical practice. J Acad Nutr Diet. (2016) 116:1178–86. 10.1016/j.jand.2016.02.013 27083988

[8] Office for Human Research Protections. Coded Private Information or Specimens Use in Research, Guidance. U.S. Department of Health & Human Services. (2008). Available online at: https://www.hhs.gov/ohrp/regulations-and-policy/guidance/research-involving-coded-private-information/index.html (accessed February 21, 2022).

[9] *IBM SPSS Statistics for MacIntosh Version 26.0 [computer program]*. Chicago, IL: SPSS Inc (2019). 10.4324/9780429056765-3

[10] Lewis SL Wright L Arikawa AY Papoutsakis C. Etiology intervention link predicts resolution of nutrition diagnosis: a Nutrition Care Process outcomes study from a Veterans' health care facility. J Acad Nutr Diet. (2021) 121:1831–40. 10.1016/j.jand.2020.04.015 32732152

[11] Chui T-K Proano G V Raynor HA Papoutsakis C. A Nutrition Care Process audit of the National Quality Improvement dataset: supporting the improvement of data quality using the ANDHII Platform. J Acad Nutr Diet. (2020) 120:1238–48. 10.1016/j.jand.2019.08.174 31668603

[12] Maduri C Sabrina Hsueh PY Li Z Chen CH Papoutsakis C. Applying contemporary machine learning approaches to nutrition care real-world evidence: findings from the National Quality Improvement data set. J Acad Nutr Diet. (2021) 121:2549–59. 10.1016/j.jand.2021.02.003 33903081

[13] Centers, for Medicare & Medicaid Services. Decision Memo for Medical Nutrition Therapy Benefit for Diabetes & ESRD (CAG-00097N). Available online at: https://www.cms.gov/medicare-coverage-database/details/nca-decision-memo.aspx?NCAId=53&fromdb=true (accessed June 1, 2022).

[14] Desroches S Lapointe A Galibois I Deschenes S-M Gagnon M-P. Psychosocial factors and intention to use the nutrition care process among dietitians and dietetic interns. Can J Diet Pract Res. (2014) 75:e335–41. 10.3148/75.1.2014.e335 24606960

[15] Gardner-Cardani J Yonkoski D Kerestes J. Nutrition Care Process implementation: a change management perspective. J Am Diet Assoc. (2007) 107:1429–33. 10.1016/j.jada.2007.05.017 17659912

[16] O'Sullivan TA Lo J Vivanti A. Predictors of Nutrition Care Process and Terminology use, applicability and importance within Asia-Pacific dietitians. Nutr Diet. (2019) 76:455–61. 10.1111/1747-0080.12467 30182523

[17] Memmer D. Implementation and practical application of the Nutrition Care Process in the dialysis unit. J Ren Nutr. (2013) 23:65–73. 10.1053/j.jrn.2012.01.025 22525801

[18] Ichimasa A. Review of the effectiveness of the Nutrition Care Process. J Nutr Sci Vitaminol. (2015) 61(Suppl):S41–3. 10.3177/jnsv.61.S41 26598881

[19] Lacey K Pritchett E. Nutrition Care Process and model: ADA adopts road map to quality care and outcomes management. J Am Diet Assoc. (2003) 103:1061–72. 10.1016/S0002-8223(03)00971-4 12891159

[20] Ansu V Papoutsakis C Gletsu-Miller N Spence LA Kelley K Woodcock L. Nutrition care practice patterns for patients with COVID-19 – a preliminary report. J Parenter Enteral Nutr. (2021) 45:1774–8. 10.1002/jpen.2106 33728687 PMC8250241

