# Supplementary material for: Documentation of the evidence-diagnosis link predicts nutrition diagnosis resolution in the Academy of Nutrition and Dietetics' diabetes mellitus registry study: A secondary analysis of Nutrition Care Process outcomes
Source: Front Nutr. 2023 Mar 9;10:1011958. doi: 10.3389/fnut.2023.1011958 (PMC10034103; doi:10.3389/fnut.2023.1011958)
Supplement: Supplementary file 1 [file Data_Sheet_1.docx]

**Supplementary Material**

Distribution of top-five Nutrition Care Process (NCP) terms (*n*=9,020) used in NCP steps in the Diabetes Registry dataset for all patients (*n*=564)

| **NCP term used in NCP step**^a^ | **NCP terminology domain**^b^ | **No. of NCP terms** | **%** |
| --- | --- | --- | --- |
| **Total** |  | 9,020 |  |
| **Nutrition Assessment** (*n*=1,756) |  |  |  |
| Glycosylated hemoglobin measurement | Biochemical Data | 296 | 16.86 |
| Body mass index | Anthropometric Measurements | 104 | 5.92 |
| Glucose, fasting | Biochemical Data | 73 | 4.16 |
| Knowledge/skill level - disease/condition | Food/Nutrition-Related History | 59 | 3.36 |
| Total carbohydrate intake | Food/Nutrition-Related History | 55 | 3.13 |
| **Nutrition Diagnosis** (*n*=2,749) |  |  | |
| **Problem** (*n*=773) |  |  | |
| Excessive carbohydrate intake | Intake | 284 | 36.74 |
| Food and nutrition related knowledge deficit | Behavioral-Environmental | 86 | 11.13 |
| Excessive energy intake | Intake | 80 | 10.35 |
| Inconsistent carbohydrate intake | Intake | 51 | 6.60 |
| Altered nutrition-related laboratory values | Clinical | 42 | 5.43 |
| **Etiology** (*n*=789) |  |  | |
| Food and nutrition related knowledge deficit | Behavioral-Environmental | 447 | 56.65 |
| Disordered eating pattern | Behavioral-Environmental | 87 | 11.03 |
| Excessive energy intake | Intake | 28 | 3.55 |
| Uncertainty how to apply nutrition knowledge | Behavioral-Environmental | 25 | 3.17 |
| Lack of prior nutrition related education | Behavioral-Environmental | 16 | 2.03 |
| **Signs & Symptoms** (*n*=1,187) |  |  | |
| Glycosylated hemoglobin measurement | Biochemical Data | 219 | 18.45 |
| Glucose, fasting | Biochemical Data | 112 | 9.44 |
| Total carbohydrate intake | Intake | 77 | 6.49 |
| Body weight | Anthropometric Measurements | 76 | 6.40 |
| Body mass index | Anthropometric Measurements | 58 | 4.89 |
| **Nutrition Intervention** (*n*=2,525) |  |  | |
| Nutrition relationship to health/disease | Nutrition Education | 296 | 11.70 |
| Other nutrition education | Nutrition Education | 262 | 10.36 |
| Recommended nutrition modifications | Nutrition Education | 255 | 10.08 |
| Priority modifications, nutrition education | Nutrition Education | 155 | 6.13 |
| Other application of nutrition education | Nutrition Education | 137 | 5.43 |
| **Nutrition Monitoring & Evaluation** (*n*=1990) |  |  | |
| Glycosylated hemoglobin measurement | Biochemical Data | 347 | 17.44 |
| Glucose, fasting | Biochemical Data | 145 | 7.29 |
| Body weight | Anthropometric Measurements | 119 | 5.98 |
| Total carbohydrate intake | Food/Nutrition-Related History | 115 | 5.78 |
| Glucose, casual | Biochemical Data | 99 | 4.97 |
| ^a,b^ The NCPT used in this dataset is the 2015 version.  NCP=Nutrition Care Process  NCPT=Nutrition Care Process Terminology | | | |
